# The Therapeutic Potential of Stem Cells in Depression

**DOI:** 10.3390/ijms26178306

**Published:** 2025-08-27

**Authors:** Lidia Jurczenko, Alina Semeniuk, Jerzy Waldemar Leszek

**Affiliations:** 1 4th Military Clinical Hospital, ul. Rudolfa Weigla 5, 50-981 Wrocław, Poland; 2 Lower Silesian Center for Oncology, Pulmonology and Hematology, Grabiszyńska 105, 53-439 Wrocław, Poland; semeniukalina2000@gmail.com; 3 Department of Psychiatry, Wroclaw Medical University, 50-367 Wrocław, Poland; jerzy.leszek@umw.edu.pl

**Keywords:** major depressive disorder (MDD), treatment-resistant depression, stem cell therapy, mesenchymal stem cells, neural stem cells, exosomes, neurogenesis, neuroinflammation, oxidative stress, brain-derived neurotrophic factor

## Abstract

Major depressive disorder (MDD) is a prevalent and disabling psychiatric condition with limited treatment options for patients who are resistant to conventional pharmacological and psychotherapeutic interventions. Stem cell (SC)-based therapies have emerged as a promising experimental approach, offering multifaceted mechanisms of action including neurogenesis, immunomodulation, antioxidative protection, and neuromodulation. This narrative review synthesizes current evidence from preclinical studies and early-phase clinical trials on the efficacy of mesenchymal stem cells (MSCs), neural stem cells (NSCs), and induced pluripotent stem cells (iPSCs) in alleviating depressive-like behaviors. Mechanistic insights include enhanced hippocampal neurogenesis, modulation of the brain-derived neurotrophic factor (BDNF)–TrkB pathway, attenuation of neuroinflammation through microglial polarization, and restoration of serotonergic signaling via peripheral-to-central pathways such as via the vagus nerve. In addition, the therapeutic potential of extracellular vesicles (EVs) and intranasal administration as non-invasive delivery strategies is discussed. While animal and first preclinical studies suggest potential benefit, significant translational barriers remain, including issues of scalability, long-term safety, and ethical considerations. Further rigorous studies are needed to validate stem-cell-based therapies as viable treatments for MDD.

## 1. Introduction

### 1.1. Major Depressive Disorder (MDD): Definition and Pathophysiology

Major depressive disorder (MDD) is the most common psychiatric illness globally, affecting approximately 3.8% of the population and up to 5.7% of those aged over 60 years [1]. Women are about 50% more likely to be affected than men. MDD ranks among the leading causes of disability worldwide and is projected to become the second most prevalent disease by 2030 [1].

MDD is defined by a persistent low mood, loss of interest, lack of sleep, and cognitive and physical symptoms that impair daily functioning. In severe cases, it may lead to suicide [2]. The condition also increases the risk of chronic diseases such as cardiovascular disease, stroke, and dementia.

Most patients with MDD respond well to pharmacological treatment, even though complete remission is not achieved in around 10–30% of cases [3].

In addition, most common antidepressants have a number of side effects such as weight gain, agitation, dry mouth, sedation, hypotension, and sexual dysfunction. These events lead to lower compliance and a lower remission rate [3].

It is now widely accepted that major depressive disorder (MDD) has a multifactorial etiology, involving a complex interplay of biological, genetic, psychological, and socio-environmental factors. Earlier hypotheses primarily attributed depression to disruptions in monoaminergic neurotransmitters such as dopamine, serotonin, and norepinephrine. However, more recent theories emphasize the dysregulation of broader neuroregulatory systems and neural circuits, with alterations in neurotransmitter levels viewed as secondary manifestations of these underlying disturbances [4,5].

Importantly, inhibitory neurotransmitters such as gamma-aminobutyric acid (GABA), as well as antagonists of NMDA receptors, have demonstrated antidepressant potential, indicating a broader neurochemical basis for mood regulation. Dysregulation of hormonal systems, including growth hormones and thyroid hormones, has also been implicated in the development of mood disorders. Furthermore, early-life trauma and chronic psychosocial stress are well-established risk factors for MDD [2]. Genetic contributions have been supported by twin studies, which consistently show a heritable component in the susceptibility to depression [2]. Furthermore, neuroimaging studies in patients with depression have consistently shown a reduced volume in key cortical and limbic regions, particularly the prefrontal cortex (PFC) and hippocampus, suggesting neuronal atrophy associated with illness duration and treatment latency [6].

The diagnosis of major depressive disorder (MDD) often begins with the identification of somatic symptoms, as patients commonly present with non-specific complaints such as headaches, back pain, or gastrointestinal disturbances. Nearly half of affected individuals do not report a depressed mood directly and may instead be referred due to social withdrawal or functional decline. Among the available screening tools, the Patient Health Questionnaire 9 (PHQ-9) is widely used for diagnosis and monitoring, with a score of ≥10 indicating a possible depressive disorder. Other commonly employed clinician- and self-rated instruments include the Hamilton Rating Scale for Depression (HAM-D) and the Montgomery–Åsberg Depression Rating Scale (MADRS). Routine assessment for suicidal ideation is essential at every clinical encounter due to the potentially life-threatening nature of depressive disorders [2].

A variety of strategies are currently available for the treatment of major depressive disorder (MDD), including pharmacological therapies, psychotherapy, interventional techniques, and lifestyle modifications. First-line treatment typically involves antidepressant medication, psychotherapy, or a combination of both. Evidence suggests that combined treatment is generally more effective than either modality alone [7].

In cases of severe, treatment-resistant, or life-threatening depression, interventions such as electroconvulsive therapy (ECT) and transcranial magnetic stimulation (TMS) have demonstrated superior efficacy compared to standard treatments [8].

If left untreated, depressive episodes in major depressive disorder (MDD) typically persist for 6 to 12 months. Approximately two-thirds of individuals with MDD experience suicidal ideation, and 10–15% ultimately die by suicide. MDD is a chronic and recurrent illness, with a recurrence rate of approximately 50% after the first episode, 70% after the second, and up to 90% after the third [3]. Overall, about 30% of patients with depressive disorder are considered to be treatment-resistant under standard clinical definitions. Additionally, around 12% of patients initially diagnosed with MDD later develop bipolar disorder [9].

The prognosis tends to be more favorable in patients with mild episodes, no psychotic features, good treatment adherence, strong social support, and good premorbid functioning [3]. Conversely, the prognosis is poorer in individuals with comorbid psychiatric or personality disorders, a history of multiple hospitalizations, or late-onset depression.

Despite the available treatment options, the high recurrence rate, risk of chronicity, and treatment resistance in a significant subset of patients underscore the need for novel therapeutic approaches. Among the emerging strategies, stem-cell-based therapies represent a promising avenue, offering the potential to modulate neurogenesis, reduce neuroinflammation, and restore impaired neural circuits associated with MDD.

### 1.2. Stem Cells (SCs)

Stem cells (SCs) are undifferentiated cells with the capacity for self-renewal and the potential to differentiate into various cellular lineages. Those properties allow SCs to serve as a cellular reservoir for development, tissue repair, and regeneration across different biological systems. Their potential for regenerative medicine, disease modeling, and drug discovery has garnered significant interest in the biomedical sciences. SCs influence host tissue via direct cell replacement and paracrine mechanisms, making them promising tools in translational research. The key properties of stem cells are summarized in Table 1.

#### 1.2.1. Classification of Stem Cells

Various classes of stem cells—including neural stem cells (NSCs), mesenchymal stem cells (MSCs), human embryonic stem cells (hESCs), and induced pluripotent stem cells (iPSCs)—have been extensively studied for their capacity to enhance neuroplasticity, modulate neuroinflammatory responses, and regulate neurogenesis.

#### 1.2.2. Neural Stem Cells (NSCs)

NSCs are multipotent stem cells that can differentiate into the three primary neural cell types: neurons, astrocytes, and oligodendrocytes. In the adult mammalian brain, NSCs are primarily localized in two neurogenic niches: the subgranular zone (SGZ) of the hippocampal dentate gyrus and the subventricular zone (SVZ) lining the lateral ventricles. Research suggests that neurogenesis in the hippocampus—an area critical for learning and memory—can be modulated by environmental, pharmacological, and cellular interventions [10]. Neural stem cells (NSCs) exhibit population-specific responses to neurogenic stimuli and pathological conditions, with at least two distinct subtypes identified [11,12,13]. One population comprises radial glia-like cells expressing glial fibrillary acidic protein (GFAP), which contribute to ongoing neurogenesis under physiological conditions or following the ablation of proliferating cells [11]. In contrast, the second population consists of horizontally oriented NSCs that are highly responsive to mitogenic signals but progressively decline or become depleted with age [13]. This age-associated reduction in horizontal NSCs is closely linked to decreased neurogenic capacity in the aging brain.

Studies in rodent models have shown that the population of stem cells in the dentate gyrus (DG) remains localized to the SGZ but tends to lose mitotic potential over time, likely entering a state of cellular senescence [14]. Although conclusive evidence is still lacking, the reduced responsiveness of these NSCs to neurogenic stimuli may indicate a progressive decline in their proliferative capacity [14]. As the mitotic ability diminishes, the proportion of actively dividing stem cells decreases, limiting the brain’s innate regenerative potential.

#### 1.2.3. Mesenchymal Stem Cells (MSCs)

Mesenchymal stem/stromal cells (MSCs) are multipotent progenitor cells capable of differentiating into mesodermal lineages such as osteoblasts, chondrocytes, and adipocytes. Their ability to modulate immune responses and secrete trophic factors has positioned MSCs as attractive candidates for regenerative medicine and immunotherapy [15]. MSCs can be isolated from diverse tissues, including bone marrow, adipose tissue, umbilical cord blood, and muscle, making them broadly accessible [16].

#### 1.2.4. Immunomodulatory and Neurotrophic Functions

MSCs exert their immunomodulatory effects through both direct cell-to-cell interactions and the secretion of bioactive molecules, including interleukin-10 (IL-10) and transforming growth factor-beta (TGF-β) [17]. They inhibit B-cell differentiation, particularly the terminal maturation into plasma cells, by releasing humoral factors that downregulate key transcriptional regulators such as Blimp-1. This suppression reduces immunoglobulin production and has been demonstrated in both in vitro and in vivo studies [18].

Importantly, MSCs also play a neuroprotective role by enhancing hippocampal neurogenesis and reducing neuroinflammation in rodent models of neurological dysfunction [19]. A key mechanism involves the secretion of brain-derived neurotrophic factor (BDNF), a neurotrophin critical for neuronal development, survival, and synaptic plasticity. BDNF signals through tropomyosin receptor kinase B (TrkB) and the p75 neurotrophin receptor, promoting synaptogenesis and connectivity. Deficits in BDNF or TrkB have been linked to impaired hippocampal function and structural atrophy [20,21].

#### 1.2.5. Comparative Characteristics: UC-MSCs vs. BM-MSCs

Studies comparing umbilical-cord-derived MSCs (UC-MSCs) and bone-marrow-derived MSCs (BM-MSCs) have revealed important functional differences. UC-MSCs exhibit a heightened ability to secrete PGE2, IL-6, and immune checkpoint molecules such as PD-L1 and PD-L2, particularly under inflammatory conditions. In contrast, BM-MSCs are more responsive to IFN-γ stimulation and produce greater amounts of IDO [22]. Functionally, UC-MSCs have shown superior immunosuppressive effects, including enhanced regulatory T-cell (Treg) promotion and reduced Th17 responses, which may offer advantages in treating inflammatory disorders [17].

MSCs also downregulate pro-inflammatory cytokines such as IL-6, TNF-α, and IL-1β, while upregulating anti-inflammatory cytokines like IL-10 and TGF-β, thereby creating a more favorable immune environment [23,24]. This property has been particularly beneficial in models of neuroinflammation, such as those induced by lipopolysaccharide (LPS), where MSC transplantation reduced oxidative stress and mitigated neural damage [25].

#### 1.2.6. Clinical Relevance and Regulatory Framework

UC-MSCs are increasingly recognized for their therapeutic advantages, including their ethical ease of sourcing, high expansion capacity, and lower immunogenicity. UC-MSCs have demonstrated therapeutic potential in systemic lupus erythematosus, graft-versus-host disease (GVHD), and inflammatory bowel disease. Clinical trials report favorable safety profiles and emerging evidence of efficacy in modulating pathological immune responses [26].

In the European Union, UC-MSCs are classified as Advanced Therapy Medicinal Products (ATMPs) under Regulation 1394/2007/EC. This designation mandates rigorous quality control and adherence to Good Manufacturing Practice (GMP) standards, which, while ensuring patient safety, introduce logistical and financial complexities for clinical deployment [26].

#### 1.2.7. Human Embryonic Stem Cells (hESCs)

Human embryonic stem cells (hESCs) are pluripotent cells derived from the inner cell mass of pre-implantation blastocysts. These cells exhibit unlimited self-renewal and the ability to differentiate into any somatic cell lineage, offering considerable promise for regenerative medicine, especially in neurodegenerative and psychiatric disorders [27]. Under specific culture conditions, hESCs can generate neural progenitors and functional neurons, which have demonstrated beneficial effects in preclinical models of neurological diseases [28].

An important aspect of hESC biology is the secretion of exosomes—nano-sized extracellular vesicles carrying signaling proteins, microRNAs, and lipids—that can influence recipient cells. These exosomes play roles in promoting proliferation, modulating inflammation, and enhancing tissue repair, thus extending the therapeutic potential of hESCs beyond direct cell replacement [16,23].

Despite their potential, the application of hESCs in clinical settings raises ethical and technical challenges. Ethical concerns stem from the destruction of human embryos, which remains a point of controversy and regulation in many jurisdictions. In addition, the high proliferative potential of hESCs poses a risk of teratoma formation if differentiation is incomplete or poorly controlled [29]. Consequently, precise differentiation protocols and stringent quality control measures are crucial for ensuring the safety and effectiveness of hESC-based therapies.

#### 1.2.8. Induced Pluripotent Stem Cells (iPSCs)

Induced pluripotent stem cells (iPSCs) are adult somatic cells that are reprogrammed into an embryonic-like pluripotent state by introducing transcription factors such as Oct4, Sox2, Klf4, and c-Myc. Like hESCs, iPSCs possess the ability to differentiate into any cell type, but without the associated ethical concerns, making them an appealing alternative for patient-specific cell therapies [29].

The iPSCs have been successfully used in neuroscience research to generate serotonergic neurons directly from human fibroblasts, using growth factors or reprogramming factors. These neurons respond to commonly used antidepressants such as citalopram and escitalopram, supporting their utility in modeling depression and screening therapeutic compounds [30,31]. Furthermore, iPSC-derived neural progenitor cells engineered to overexpress brain-derived neurotrophic factor (BDNF) have been shown to enhance neurogenesis and reverse depression-like behavior in rodent models exposed to chronic stress [32].

Similar to hESCs, iPSCs also secrete exosomes that play key roles in intercellular communication. These vesicles contain bioactive molecules that can modulate immune responses, reduce inflammation, and promote neuronal repair [16]. However, challenges remain, including the risk of genomic instability due to reprogramming, potential tumorigenicity, and variable differentiation efficiency. Therefore, ongoing efforts focus on improving reprogramming techniques and ensuring the consistency and safety of iPSC-derived therapies [29].

Summary of the main features of different SCN stem cells is presented in Table 2.

### 1.3. Mechanisms of Action of Stem Cell (SC)-Based Therapies in Depression

Stem cell (SC)-based therapies exert antidepressant effects through several interrelated mechanisms. One of the most consistent findings is their ability to enhance hippocampal neurogenesis, which is frequently disrupted in depression, by stimulating the proliferation and differentiation of neural precursors in the dentate gyrus [33]. In addition, mesenchymal stem cells (MSCs) have been shown to enhance serotonergic activity in the dorsal raphe nucleus (DRN) under stress conditions. By modulating BDNF–TrkB pathways, MSCs restore serotonin synthesis and exert antidepressant effects, while MSC-derived exosomes further promote the production of β-endorphin, dopamine, and serotonin, possibly via the vagus nerve–brain axis [34].

Another important mechanism involves anti-inflammatory and antioxidant actions. MSC therapy reduces oxidative stress, a key feature of major depressive disorder (MDD), by generating antioxidants such as hydrogen sulfide (H2S), which upregulates Sirt1 and modulates inflammatory cytokines [35]. Moreover, MSCs shift microglia from the pro-inflammatory M1 to the anti-inflammatory M2 phenotype, rebalancing immune signaling [36]. They also promote expansion and suppress effector T-cell activity [17].

Beyond direct cell actions, non-cell-autonomous mechanisms also play a role. Engineered MSCs secrete neurotrophic factors such as BDNF, VEGF, FGF2, and CNTF, and when implanted into the ventricles they provide both behavioral and neurogenic benefits. Similarly, MSC-derived exosomes, including those carrying miR-26a, enhance neuronal survival and proliferation, underscoring their potential as a cell-free therapeutic platform [36].

Finally, modulation of the hypothalamic–pituitary–adrenal (HPA) axis has been explored, since this system is often dysregulated in MDD. While SC transplantation has not significantly altered CRH or dopaminergic neuron activity in preclinical models [37], the possibility that SCs influence HPA-related genes such as FKBP51 and BDNF remains an open question for future research.

## 2. Aim of This Study

This narrative review aims to provide a comprehensive overview of current knowledge regarding the therapeutic potential of stem-cell-based interventions in major depressive disorder (MDD). By synthesizing evidence from both preclinical models and early-phase clinical studies, we seek to elucidate the underlying mechanisms of action and summarize the initial outcomes associated with stem cell therapies for depression.

## 3. Results

### 3.1. Preclinical Efficacy in Animal Models

Preclinical research demonstrates that stem-cell-based therapies—particularly those involving mesenchymal stem cells (MSCs)—hold considerable promise for treating depression. Studies using various animal models of depression, including chronic stress paradigms and treatment-resistant strains like Wistar-Kyoto rats, have shown that MSCs and related approaches such as cell encapsulation and extracellular vesicles (EVs) can significantly reduce depressive-like behaviors.

One notable study by Kin et al. revealed that encapsulated MSCs (eMSCs), when implanted into the lateral ventricle of Wistar-Kyoto rats, not only attenuated depressive-like behaviors but also promoted endogenous neurogenesis in the subventricular zone and hippocampal dentate gyrus [33]. These effects were attributed to the sustained secretion of neurotrophic factors and activation of associated signaling pathways. In contrast, non-encapsulated MSCs or eMSCs implanted outside the neurogenic niches failed to produce similar benefits, underscoring the importance of both the delivery method and anatomical targeting.

**Figure 1:**
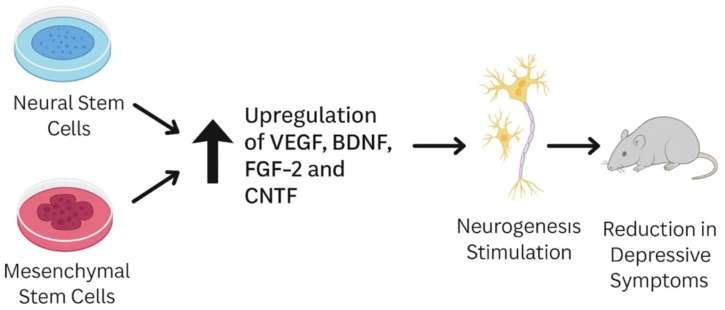
Schematic showing how MSCs and NSCs alleviate depressive symptoms in animal models by upregulating VEGF, FGF-2, CNTF, and BDNF signaling, typically reduced in depression.

Furthermore, Costa-Ferro et al.conducted a study on rats in which they demonstrated that bone marrow mononuclear cell transplantation prevents the development of depression- and anxiety-like behavior triggered by chronic mild stress [38]. The treatment inhibited the upregulation of pro-inflammatory markers HMGB1 and IL-1β in the hippocampus while enhancing the expression of the anti-inflammatory factor BDNF in the same region [39]. A schematic illustration of this experimental process is presented in Figure 1.

Furthermore, adipose-derived MSCs and human umbilical-cord-derived MSCs (hUC-MSCs) have both been shown to suppress microglial activation and promote a shift toward an anti-inflammatory phenotype. In a study conducted by Wang et al., depressive behaviors in rats, along with elevated pro-inflammatory cytokines and morphological abnormalities in the hippocampus, were reversed within four weeks following the administration of hUC-MSCs [23]. Furthermore, the study by Huang et al. suggests a potential antidepressant mechanism linked to microglial polarization induced by anti-inflammatory agents [39].

MSC-derived EVs increased hippocampal neuroblasts and dendritic complexity, further contributing to antidepressant-like effects [32]. Also, the other form, administered systemically or intracerebrally, has been shown to reverse stress-induced behavioral deficits, reduce inflammatory markers, and promote hippocampal plasticity [40].

Interestingly, MSCs may also modulate serotonergic activity independently of inflammation [30]. Some studies suggest that MSC-secreted BDNF can activate serotonin neurons in the dorsal raphe nucleus via vagus-nerve signaling pathways [37,41]. Additionally, oxidative stress reduction, evidenced by decreased 8-OHdG levels in BMMC-treated animals, represents another mechanism through which stem cell therapies may confer neuroprotection [42].

Studies further indicate that mesenchymal stem cells (MSCs), whether administered systemically or intracerebrally, reverse stress-induced behavioral deficits, reduce inflammatory markers, and promote hippocampal plasticity [43].

Combination therapies show even greater promise. Kigawa et al. demonstrated that co-administration of MSCs with first-line antidepressants such as sertraline results in synergistic effects, leading to enhanced efficacy in treatment-resistant depression models [44].

Another promising study conducted by Zhang et al. investigated the efficacy of human umbilical-cord-derived mesenchymal stem cells (hUC-MSCs) in a myocardial infarction (MI)-induced depression model. Following left anterior descending (LAD) coronary artery ligation, mice exhibited both cardiac dysfunction and depression-like behaviors. Intravenous administration of hUC-MSCs led to improvements in both cardiac function and mood-related symptoms [45].

Mechanistically, the treatment downregulated Jmjd3—a histone demethylase involved in inflammatory signaling—and modulated microglial polarization by suppressing M1 markers (CD86, iNOS) and upregulating M2 markers (CD206, Arg-1) in the hippocampus, prefrontal cortex, and hypothalamus [23]. This was accompanied by the reduced expression of inflammatory cytokines in both central and peripheral tissues. While Jmjd3’s role in depression remains complex, the study highlights it as a potential target in MI-induced neuroinflammation [46]. Limitations included the absence of MSC tracking and unresolved questions regarding direct causality between Jmjd3 modulation and microglial polarization [45].

Shwartz et al. explored MSCs engineered to overexpress excitatory amino acid transporter 1 (EAAT1) in Flinders Sensitive Line (FSL) rats, a genetic model of depression. These MSC EAAT1 cells were stereotactically injected into the lateral ventricle and subsequently migrated to the hippocampus. They significantly increased EAAT1 and BDNF expression, promoted astrocytic function, and reduced glutamate toxicity. Behavioral assays, including the Forced Swim Test (FST), Sucrose Preference Test (SPT), and Novelty-Suppressed Feeding Test (NSFT), indicated pronounced antidepressant-like effects, affirming the utility of MSCs in modifying excitatory neurotransmission and neuroplasticity [47].

In a comprehensive review, Kot et al. elaborated on how MSCs enhance adult hippocampal neurogenesis and exert antidepressant-like effects predominantly via secreted factors rather than direct cell integration [48]. Intracerebroventricular administration of MSCs stimulated neurogenesis and improved mood-related behaviors, while intra-hippocampal transplantation enhanced neurogenesis alone [49].

The MSC secretome, rich in PGE2, TGF-β, and other bioactive molecules, modulates neuroinflammation by suppressing mast cell activity. Given that mast cells synthesize serotonin and initiate early neuroinflammatory responses, their modulation is likely critical to MSC-mediated antidepressant effects [50]. Nonetheless, mast cell activation is context-dependent and can yield both neuroprotective and detrimental outcomes [41].

Finally, Sachdeva et al. emphasized the potential of stem cells, particularly MSCs, as non-pharmacological interventions for stress-induced depression. MSCs reduce neuroinflammation by promoting the transition of microglia from the M1 to the M2 phenotype and by upregulating IL-10 and TGF-β. Through paracrine signaling and exosome release, MSCs enhance BDNF and VEGF levels, support synaptic plasticity, and restore mitochondrial function while reducing oxidative stress [51]. Key findings from studies on animal models are summarized in Table 3.

To date, evidence supporting the efficacy of stem cell (SC) therapy in humans remains limited but promising. One notable study was conducted by Smulevich et al., who investigated the therapeutic potential of umbilical-cord blood cells in women with treatment-resistant depression. Participants received four weekly injections of 250 million concentrated umbilical-cord blood cells. A control group received placebo injections. While both groups demonstrated a reduction in depressive symptoms and hypothymia, the treatment group showed a delayed yet more pronounced improvement in treatment resistance and depressive severity. Additionally, long-term cognitive improvement was observed in the stem-cell-treated group [52]. These findings suggest that umbilical cord blood cells may help to overcome treatment resistance in depressive disorders. However, further preclinical studies are needed to clarify whether therapeutic effects depend on neural engraftment, the stage of stem cell differentiation, or interactions with concurrent antidepressant treatment.

As of now, two active clinical trials investigating the use of stem cells or stem-cell-derived products in depression have been registered on ClinicalTrials.gov, while one has been terminated due to a lack of funding:

NCT03265808: a phase I/II randomized, double-blind, placebo-controlled trial evaluating the safety of allogeneic human mesenchymal stem cell (MSC) infusions in 80 participants with comorbid Alcohol Use Disorder and Major Depression (AUD-MD) [53].

NCT03522545: a double-blind, placebo-controlled trial designed to assess the efficacy and tolerability of allogeneic bone-marrow-derived multipotent mesenchymal stromal cells in patients with treatment-resistant bipolar depression (TRBD). This study is ongoing and currently in the recruitment phase, with a planned enrollment of 30 participants [53].

NCT02675556: a study evaluating the safety and potential efficacy of allogeneic MSC infusions in patients with treatment-resistant depression. The trial was terminated in 2019 due to insufficient funding [53].

Although these early-phase trials reflect a modest level of clinical engagement with stem-cell-based therapies for depressive disorders, the robust and consistent findings from preclinical studies underscore the need for continued research. Further clinical investigations are essential to validate therapeutic efficacy, define optimal treatment protocols, and ensure long-term safety.

#### Delivery Strategies: Intranasal Administration

One of the major limitations in CNS-targeted stem cell therapy is the presence of the blood–brain barrier (BBB), which significantly restricts the passage of therapeutic agents [54,55]. Although stereotactic and intrathecal injections have been commonly employed for stem cell delivery to the CNS, their invasive nature restricts broader clinical application.

Intranasal administration (INA) has recently gained attention as a non-invasive, efficient method for delivering stem cells and other therapeutics to the CNS. This technique bypasses the BBB and allows direct access to the brain through the olfactory and trigeminal pathways or the cerebrospinal fluid system. INA offers several advantages over intravenous and intracranial methods: it avoids first-pass metabolism, allows repeated administration, minimizes exposure to peripheral organs, and is simpler to use [55].

Studies have shown the successful INA delivery of NSCs, MSCs, and iPSCs in models of glioma, neurodegeneration, brain injury, and chronic inflammation [40]. INA facilitates the accumulation of substances in the nasal epithelium, followed by paracellular and intracellular transport to the CNS [55]. Given its efficacy and safety profile, INA represents a promising strategy for future non-invasive stem-cell-based treatments in depression and other neurological disorders [55].

## 4. Discussion

The findings summarized in this review highlight both the mechanistic plausibility and promising preclinical efficacy of several stem cell types, particularly neuronal and mesenchymal stem cells (MSCs), in alleviating MDD.

One of the most consistently reported mechanisms is the enhancement of hippocampal neurogenesis, a process frequently impaired in MDD [49]. Animal studies, such as those by Kin K. et al. and Costa-Ferro et al. and Wang et al., indicate that MSC treatment demonstrates significant antidepressant effects in a depression model, surpassing even fluoxetine in efficacy [32,33,46].

Additionally, stem cells have been shown to promote the proliferation and differentiation of neural precursors in the dentate gyrus, supported by the upregulation of neurotrophic factors such as brain-derived neurotrophic factor (BDNF), fibroblast growth factor-2 (FGF-2), and vascular endothelial growth factor (VEGF) [33]. These growth factors are crucial for synaptic plasticity and structural repair in brain regions implicated in mood regulation, including the hippocampus and prefrontal cortex [56].

In addition to neurogenesis, MSCs possess robust immunomodulatory capabilities. By promoting microglial polarization from pro-inflammatory (M1) to anti-inflammatory (M2) states, MSCs attenuate central and peripheral inflammation—a key contributor to depression pathogenesis [23,36]. This shift is mediated by the secretion of anti-inflammatory cytokines including IL-10 and TGF-β, alongside the suppression of IL-1β and TNF-α [24]. Notably, in rodent models such as Wistar-Kyoto rats, encapsulated MSCs implanted into neurogenic regions led to sustained anti-inflammatory and behavioral improvements [33].

Oxidative stress, another pathological hallmark of MDD, is also mitigated by stem cell therapies. Studies show that MSCs increase hydrogen sulfide (H_2_S) production and upregulate Sirt1, enhancing mitochondrial resilience and reducing reactive oxygen species [24,57,58]. These antioxidative effects complement their immunomodulatory profile and contribute to the overall neuroprotective action observed in animal models.

Moreover, recent evidence points to a novel mechanism involving the gut–lung–vagus-nerve–brain axis [37] and demonstrated that adipose-derived MSCs exert antidepressant effects via the BDNF-mediated activation of serotonergic neurons in the dorsal raphe nucleus. This suggests that even peripheral administration of MSCs can influence central neurotransmission, expanding the potential of non-invasive delivery strategies.

A study conducted by Abdulmalek [15] as well as that of Guo et al. [56] indicated that the administration of stem-cell-derived exosomes and extracellular vesicles (EVs) offers an additional, cell-free therapeutic avenue. Indeed, EVs enriched with microRNAs (e.g., miR-26a) and neurotrophic factors have been shown to enhance hippocampal neurogenesis and suppress inflammation, with a reduced risk of immune rejection or tumorigenesis [56].

Preclinical studies have further demonstrated that combining MSC therapy with traditional antidepressants, such as SSRIs, results in synergistic behavioral improvement [44,48]. This reinforces the notion that stem cell therapies could serve as adjunctive treatments in cases of treatment-resistant depression (TRD).

Despite these promising outcomes, significant translational hurdles persist. First, the majority of data originate from small-animal models, which do not fully recapitulate the complexity of human depression. Differences in immune system dynamics, neural architecture, and stress responses must be considered when extrapolating results to clinical populations. Second, the long-term safety of stem cell therapies remains insufficiently characterized. Concerns such as genomic instability, ectopic differentiation, and potential tumor formation—particularly with pluripotent stem cells—necessitate continued monitoring and protocol refinement [44,51]. Third, two cited clinical trials were small, early-phase studies that lacked placebo-controlled groups and did not include long-term follow-up, limiting the strength and generalizability of their findings.

Furthermore, ethical, logistical, and regulatory constraints further complicate the path toward clinical implementation. For example, while human embryonic stem cells (hESCs) and induced pluripotent stem cells (iPSCs) offer broad differentiation potential, they raise concerns about embryo destruction and reproducibility, respectively [51].

In conclusion, stem-cell-based therapies—particularly those involving MSCs and their derivatives—represent a compelling and biologically grounded strategy for addressing the multifactorial pathophysiology of MDD. Their combined effects on neurogenesis, inflammation, oxidative stress, and neuromodulation provide a strong mechanistic rationale for further investigation. However, rigorous translational research, standardized protocols, and ethical oversight will be essential before these therapies can be integrated into clinical practice.

### Future Directions

Innovations in stem cell engineering and biotechnology may help to overcome current limitations. The development of SCs engineered to express therapeutic genes, enhance homing to damaged brain regions, or withstand hostile inflammatory environments holds significant promise [49,50].

Exosome-based therapies offer a less invasive alternative, enabling the targeted delivery of regenerative and immunomodulatory molecules across the blood–brain barrier. Advances in cell reprogramming and delivery technologies, including INA, are poised to facilitate more effective and personalized therapeutic strategies in future clinical applications.

## 5. Conclusions

While stem cell therapy represents a compelling and innovative approach to treating depression, it is not without limitations. Its multifactorial therapeutic mechanisms offer real potential to address the neurobiological complexity of MDD, especially in cases unresponsive to existing treatments. However, the field is still in its infancy, and further rigorous research is needed to confirm efficacy, clarify mechanisms of action, and ensure safety across diverse patient populations. Ethical oversight, cost-effectiveness, and regulatory harmonization will also be critical as stem-cell-based strategies move closer to clinical application. Until such challenges are met, SC therapy should remain within the domain of controlled research rather than routine clinical practice.

## Figures and Tables

**Table 1:** Fundamental properties of stem cells.

Key Property	Description
Self-renewal	Ability to proliferate while maintaining an undifferentiated state.
Multilineage differentiation	Capacity to generate various specialized cell types.
Homing	Directed migration to sites of injury or inflammation.
Immunological tolerance	Low immunogenicity that facilitates transplantation.

**Table 2:** Comparison of stem cell types.

Stem Cell Type	Origin	Differentiation Potential	Immunogenicity	Clinical Use
NSC	Neural tissue	Tripotent (neurons, astrocytes, oligodendrocytes)	Low	CNS repair
MSC	Bone marrow, adipose tissue, umbilical cord	Multipotent	Very low	Immunomodulation, neurotrophic support
hESC	Blastocyst	Pluripotent	High	Experimental applications
iPSC	Reprogrammed somatic cells	Pluripotent	Medium	Personalized medicine, disease modeling

NSC: neural stem cell, MSC: mesenchymal stem cell, hESC: human embryonic stem cell, iPSC: induced pluripotent stem cell, CNS: central nervous system.

**Table 3:** Key findings derived from animal studies.

Study/Authors	Stem Cell Type	Model	Key Outcomes
Kin et al. [33]	Encapsulated MSCs (eMSCs)	Wistar-Kyoto rats (treatment-resistant depression model)	The eMSCs implanted into the lateral ventricle alleviated depressive-like behaviors and boosted neurogenesis in the SVZ and hippocampal dentate gyrus. These effects were not observed with non-encapsulated MSCs or with eMSCs implanted outside neurogenic regions.
Costa-Ferro et al. [38]	Bone marrow mononuclear cells	Rats (chronic mild stress model)	Prevented depression/anxiety-like behavior, reduced HMGB1 and IL-1β, increased BDNF
Wang et al. [43]	Human umbilical-cord-derived MSCs (hUC-MSCs)	Rats (depression model)	Reversed depressive behavior, normalized hippocampal morphology, reduced cytokines
Kigawa et al. [44]	MSCs and sertraline	Rats (prenatal and adolescent induced depression)	Synergistic antidepressant effects
Zhang et al. [45]	hUC-MSCs	Myocardial-infarction-induced depression in mice	Improved cardiac and depressive symptoms, downregulated Jmjd3, modulated microglial polarization
Shwartz et al. [47]	MSCs overexpressing EAAT1	FSL rats (genetic depression model)	MSC-EAAT treatment in FSL rats reduced depressive-like behaviors and increased EAAT1 and BDNF expression in the hippocampus.

## Abbreviations

The following abbreviations are used in this manuscript:

MDD	Major depressive disorder
SC	Stem cell
NSC	Neural stem cell
MSC	Mesenchymal stem cell
eMSC	Encapsulated mesenchymal stem cell
hESC	Human embryonic stem cell
iPSC	Induced pluripotent stem cell
UC-MSC	Umbilical-cord-derived mesenchymal stem cell
BM-MSC	Bone-marrow-derived mesenchymal stem cell
BDNF	Brain-derived neurotrophic factor
CNTF	Ciliary neurotrophic factor
FGF-2	Fibroblast growth factor 2
VEGF	Vascular endothelial growth factor
IL-1β	Interleukin-1 beta
IL-6	Interleukin-6
IL-10	Interleukin-10
TNF-α	Tumor necrosis factor alpha
TGF-β	Transforming growth factor beta
PGE2	Prostaglandin E2
IDO	Indoleamine 2,3-dioxygenase
GABA	Gamma-aminobutyric acid
NMDA	N-Methyl-D-aspartate
DRN	Dorsal raphe nucleus
EVs	Extracellular vesicles
HPA	Hypothalamic–pituitary–adrenal (axis)
PHQ-9	Patient Health Questionnaire 9
HAM-D	Hamilton Rating Scale for Depression
MADRS	Montgomery–Åsberg Depression Rating Scale
GDS	Geriatric Depression Scale
HADS	Hospital Anxiety and Depression Scale
CNS	Central nervous system
INA	Intranasal administration
LPS	Lipopolysaccharide
FST	Forced Swim Test
SPT	Sucrose Preference Test
CRH	Corticotropin-releasing hormone
